# Treadmill Training in Patients with Parkinson’s Disease: A Systematic Review and Meta-Analysis on Rehabilitation Outcomes

**DOI:** 10.3390/brainsci15080788

**Published:** 2025-07-24

**Authors:** Elisa Boccali, Carla Simonelli, Beatrice Salvi, Mara Paneroni, Michele Vitacca, Davide Antonio Di Pietro

**Affiliations:** 1 Istituti Clinici Scientifici Maugeri IRCCS, Neurorehabilitation of the Institute of Lumezzane, 25065 Lumezzane, Italy; elisa.boccali@icsmaugeri.it; 2 Istituti Clinici Scientifici Maugeri IRCCS, Cardiac Rehabilitation of the Institute of Lumezzane, 25065 Lumezzane, Italy; carla.simonelli@icsmaugeri.it (C.S.); davide.dipietro@icsmaugeri.it (D.A.D.P.); 3 Istituti Clinici Scientifici Maugeri IRCCS, Respiratory Rehabilitation of the Institute of Lumezzane, 25065 Lumezzane, Italy; beatrice.salvi@icsmaugeri.it (B.S.); mara.paneroni@icsmaugeri.it (M.P.)

**Keywords:** Parkinson’s disease, treadmill, walking, exercise, rehabilitation, outcomes

## Abstract

**Background/Objectives**: Parkinson’s disease (PD) is a neurodegenerative disorder that impairs mobility. **Treadmill training (TT)** is a common rehabilitation strategy for improving gait parameters in individuals with PD. This systematic review evaluated the effectiveness of TT in improving motor function, walking ability, and overall functional mobility in PD patients. **Methods**: We compared TT to other forms of gait and motor rehabilitation, including conventional and robotic gait training. Trials that compared a treadmill training group with a non-intervention group were excluded from this review. We searched multiple databases for RCTs involving Parkinson’s patients until January 2025. The primary outcomes were motor function (UPDRS-III) and walking ability (6 MWT and TUG test). **Results:** We identified 285 articles; 199 were excluded after screening. We assessed the full text of 86 articles for eligibility, and 13 RCTs met the inclusion criteria. Some of them were included in the meta-analysis. The TT group showed a significant improvement in UPDRS-III scores [mean difference (MD): −1.36 (95% CI: −2.60 to −0.11)] and greater improvement in TUG performance [MD, −1.75 (95% CI: −2.69 to −0.81)]. No significant difference in walking capacity as assessed through the 6 MWT was observed [MD: 26.03 (95% CI: −6.72 to 58.77). **Conclusions:** The current study suggests that TT is effective in improving the motor symptoms and functional mobility associated with PD. Further studies are needed to develop protocols that consider the patients’ clinical characteristics, disease stage, exercise tolerance, and respiratory function.

## 1. Introduction

Parkinson’s disease (PD) is defined as a chronic, progressive, and degenerative disorder of the central nervous system due to the depletion of dopaminergic neurons in the nigrostriatal pathway [1].

In high-income countries, its incidence is estimated to be 14 per 100,000 people in the total population and 160 per 100,000 in people aged 65 and over [2]. The main symptoms of Parkinson’s disease include resting tremors and gait disturbances (freezing and instability), which can affect patients’ mobility and independence, increasing the risk of falls and compromising their quality of life [3,4,5].

The characteristics and details of gait disorders have been studied extensively in recent years [6], and various rehabilitation strategies have been proposed to slow down and reduce the impact of gait disorders on the daily lives of people with Parkinson’s disease [7], including different types of exercise and physical activity [8].

Among these, treadmill training (TT) is safe and effective in improving gait in PD patients and has been proposed as a stand-alone treatment or in combination with complementary therapies such as rhythmic auditory stimulation (RAS) [6,7,8,9].

A Cochrane review of 18 trials comparing TT with no TT (including no intervention) showed that TT may improve clinically relevant gait parameters, such as gait speed and stride length, in patients with PD. However, it failed to demonstrate a positive effect on functional capacity, such as walking distance [9]. A subsequent meta-analysis of 32 trials (*n* = 823) comparing TT with no exercise or a sham treatment showed a moderate effect on 10 m walk test (10 MWT) performance and a moderately large effect on gait speed in favor of TT [7].

However, although the efficacy of rehabilitation with treadmill training compared to placebo or no rehabilitation has been highlighted, it has not been clearly established whether treadmill training is superior to conventional and unconventional non-pharmacological treatments.

A recent review investigated the effects of TT on functional capacity in people with PD. It was shown that TT with or without weight support (at least 20 min, two to three times a week, with progressively increasing loads, for a minimum of 6 weeks) was able to improve gait outcomes in elderly Parkinson’s disease patients, but further studies are warranted [6].

The aim of this systematic review and meta-analysis of randomized controlled trials (RCTs) was to assess the effectiveness of TT in improving motor symptoms, walking capacity, and functional mobility. Thus, we conducted a review on treadmill activity compared to active rehabilitation activities (TT vs. other rehabilitation methods), including conventional or robotic gait training, and excluded trials that compared treadmill training groups with non-intervention groups.

## 2. Materials and Methods

This study conforms to all Preferred Reporting Items for Systematic Reviews and meta-analysis guidelines and reports the required information accordingly (Supplementary Materials). No prior registration was planned for this study.

### 2.1. Data Sources and Search Strategies

The MEDLINE, EMBASE, CINAHL, Scopus, and Web of Science databases were searched without language restriction from their inception until 31 January 2025. We also checked the bibliographies of the retrieved articles for additional studies.

We created a PICO strategy for the search, where P is Parkinson’s disease patients; I is rehabilitation of gait and walking by treadmill training; C is other rehabilitation treatments dedicated to gait and walking (e.g., conventional gait training, robot-assisted gait walking, etc.); and O is walking outcomes. We used the following query: ((treadmill OR ambulatory OR gait) AND (Parkinson) NOT (pediatrics OR musculoskeletal OR posture OR postural OR animal OR amputee OR trunk OR knee OR hip OR spasticity OR heart OR blood OR cardiac)); filter: randomized controlled trial.

### 2.2. Patients and Programs

We included all RCTs that included adults with a clinical diagnosis of PD [10] at any stage of the disease who were in a chronic and stable phase. We included trials of training programs with the following characteristics:

- Inpatient or outpatient or home- or community-based;
- Had at least one study group that underwent TT, either with or without adjunctive treatments (e.g., rhythmic auditory stimulation (RAS), weight-bearing support systems, belts, etc.);
- Had at least one study group that underwent another type of gait and walking rehabilitation, such as conventional or robotic gait training.

We excluded all trials that compared treadmill training groups with non-intervention groups or that compared two types of TT with different characteristics. We also excluded trials that compared TT with other types of training not specifically related to walking (such as cycling endurance training). We also excluded trials with mixed populations.

All interventions consisting of any type of robot-assisted gait training (RAGT), even when performed on a treadmill, were excluded. Instead, all TT interventions that included treadmill walking plus a passive safety or body weight support system were included in the analysis. Trials comparing TT with RAGT were included and categorized as described below.

To facilitate the interpretation and discussion of the results, the studies were classified according to the rehabilitation programs administered to the control groups as follows:

- Robot-assisted rehabilitation, performed with any robotic walking aid device;
- Conventional rehabilitation, defined as any rehabilitation treatment that included gait and walking exercises, including dance, that did not require any of the above technologies. RAS delivered via headphones during walking was considered equivalent to treatments in which the RAS was delivered verbally by the physiotherapist and was therefore included with conventional treatments.

### 2.3. Outcome Measures

Basing on the European Physiotherapy Guidelines for Parkinson’s Disease [11], we included all trials that focused the analysis on at least one of the following key outcomes: (i) motor symptoms, using part III of the Unified Parkinson Disease Rating Scale (UPDRS-III) [12]; (ii) walking capacity, using the 6 min walk test (6 MWT) [13]; and (iii) functional mobility, using the Timed Up and Go (TUG) test [14].

### 2.4. Data Collection and Analysis

Two reviewers (EB and CS) conducted the first step of the meta-analysis. The main reviewer (EB) searched the bibliographic databases, screening all titles and/or abstracts for the inclusion criteria, and then retrieved abstracts and/or full texts of all potentially eligible studies and maintained records of all the studies that did not meet the inclusion criteria, including the reasons for their exclusion. A second reviewer (CS) validated the eligible articles, and disagreements between the reviewers about eligibility were resolved by discussion and consensus. A third reviewer (MP) adjudicated when a consensus could not be reached by the two main reviewers.

All recorded data of the final selected trials were checked for accuracy. The main reviewer (EB) separated the original full-text papers that were selected for inclusion in the meta-analysis from the other articles and entered their data into a dedicated electronic database (Excel software, Microsoft, Redmond, WA, USA). The second reviewer (CS) independently extracted data from the same trials.

The following information was included: study type; study participant characteristics including age, sex, and baseline UPDRS score and/or Hoehn and Yahr scale score [15]; full description of training programs (type of training, duration, frequency, intensity, and characteristics) and setting; brief description of any other adjunctive treatment given during the study period; and motor function measures, gait analysis results, and associated outcomes. If a trial reported multiple group comparisons (e.g., high-intensity TT vs. low-intensity TT vs. conventional gait rehabilitation), all the treatment groups, including TT, were combined into a virtual intervention group, and this group was compared with the group receiving other forms of gait rehabilitation. If a non-intervention group was present, it was not included in the analysis of the results.

The researchers assessed the risk of bias in the trials using the Cochrane Collaboration tool for assessing the risk of bias in RCTs (RoB 2) [16].

The domains included in the RoB 2 cover the following biases: bias due to the randomization process, bias due to deviations from the intended interventions, bias due to missing outcome data, bias in the measurement of the outcome, and bias in the selection of the reported outcome. For each domain, the RoB 2 tool consists of a series of signaling questions and an algorithm that maps the answers to these questions to a proposed judgment.

Using the RoB2 Excel tool, the same reviewers (EB and CS) independently answered the tool’s signaling questions for each study; they then reviewed the judgment provided by the tool’s algorithm and confirmed or modified it according to their judgment, providing a final judgment for each domain: low risk, some concerns, or high risk of bias. These judgments were summarized in two dedicated tables and are available in the Supplemental Materials (Tables S1 and S2). The two tables were then compared, and disagreements were resolved by discussion. A third author (MP) was available to adjudicate when a consensus could not be reached. Given that the meta-analysis only included 13 studies, funnel plot analysis and other methods for detecting publication bias (e.g., Egger’s test) were considered unreliable and not informative in this context.

### 2.5. Statistical Analysis

Statistical analysis was performed using R statistical software (v3.1.3; R Core Team 2015 [17]. All data were extrapolated from the full texts of the studies. For each outcome (motor function and walking ability), we recorded the mean and standard deviation (SD) of variation from baseline to the end of the study [18].

When the SD was not available, we calculated the standard error as follows:

$$\sqrt{\frac{sd_{1}^{2}}{n_{1}}+\frac{sd_{2}^{2}}{n_{2}}}$$

Then, we estimated the SD as $\mathrm{SD}=\mathrm{SEMx}\sqrt{n}$ [19].

The weighted mean difference (MD) was calculated. Both fixed-effect and random-effect models were considered to determine the appropriate method for the meta-analysis. The decision to use either model was based on the assessment of heterogeneity using the I^2^ statistic and Q-test (a *p*-value < 0.10 for Cochran’s Q-test and/or an I^2^ statistic greater than 50%) as well as a careful evaluation of the similarity between the studies included in the analysis. As a first step, fixed-effect models were used. If significant heterogeneity between studies was found, a random-effect evaluation was performed using the Der-Simonian and Laird method approach [20]. Forest plots were used to present the results.

We also considered, for comparison, Minimal Clinically Important Difference (MCID) scores for PD patients or the general population for the primary outcomes. The MCID for UPDRS-III was estimated by Horvath [21] to be −3.25 points for detecting minimal but clinically pertinent improvement. The MCID for 6 MWT was found to be +30.5 m in a general population by Bohannon et al. [22], and the minimal detectable change (MDC) of the TUG test in people with Parkinson’s disease (that is, the smallest amount of difference in individual scores that represents true change) was reported by Huang et al. [23] to be −3.5 s.

## 3. Results

**Figure 1:**
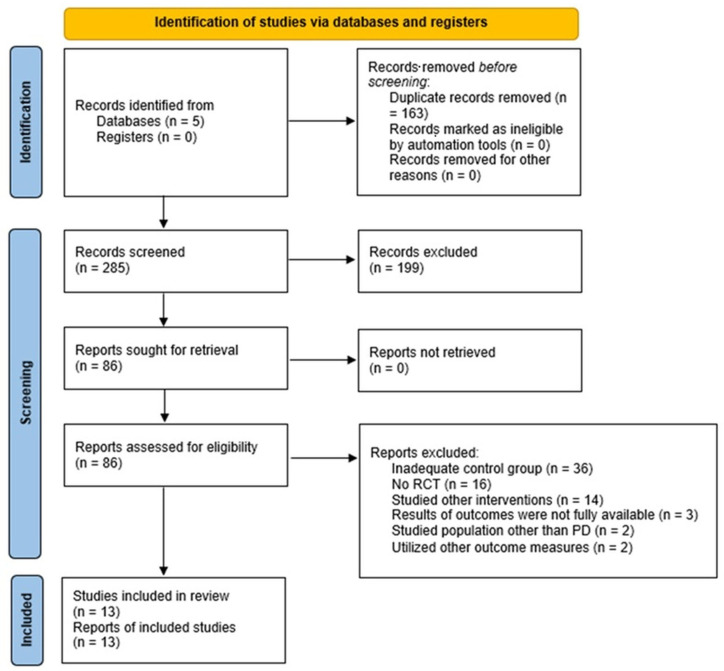
Flow diagram of the included studies.

We initially identified 285 records: 199 records were excluded after title/abstract screening, while 86 full-text articles were assessed for eligibility. Finally, 13 studies were included in the qualitative synthesis and meta-analysis (Figure 1).

The participants in the included studies were middle-aged, of normal height and weight, with a mean PD history of 6.5 years, no cognitive impairment, mild to moderate PD, and mild motor impairment. The details of the participant characteristics are shown in Table 1.

The stage of PD, as assessed by the Hoehn and Yahr scale [15], varied between studies, with the majority excluding the most severe stage 4 [24,25,26,27,28]. One study [29] included less severe PD, two included PD stages 2.5 and 3, while Picelli et al. [30] only included PD stage 3. Conversely, Capecci et al. [31] excluded the less severe PD stage 1. Rawson et al. [32] and Bello et al. [33] did not report criteria for PD severity. The details of the included studies are shown in Table 2.

## 4. Interventions

The majority of the TT programs in the studies included in this review were delivered to outpatients [24,25,29,30,31,34]. Rawson et al. [32] reported a community-based intervention, while the other studies did not specify the setting.

Among the thirteen included studies, the characteristics of the TT programs varied widely. Ten studies [25,26,27,29,30,31,32,33,34,35] used a standard TT program, while two studies [24,36] used a treadmill with a partial weight support system, and one study [28] used a curved treadmill. The duration of the TT program ranged from 4 to 24 weeks, with seven studies [24,28,30,31,34,35,36] specifically investigating the short-term effects of a one-month TT program.

The frequency of the TT sessions varied, with studies reporting sessions twice a week [25,28,32], three times a week [26,27,28,29,33,35,36], four times a week [24], or five times a week [31]. The session duration ranged from a minimum of 15 min [27] to a maximum of 60 min [32], with considerable variability between trials.

Two studies [27,33] progressively increased both the TT intensity and session duration, whereas all the other studies maintained a fixed session duration and increased the intensity throughout the training period.

Five studies [25,27,29,33,34] implemented a constant speed training protocol, while another five [28,30,31,35,36] progressively increased the speed during each session. In contrast, four studies [24,26,30,33] proposed an interval training approach, alternating work intervals (4 to 10 min) with rest or low-intensity work intervals (2 to 5 min).

In terms of TT intensity, all trials reported the initial treadmill speed and the workload progression. All the TT programs were performed at a 0% incline, except for two studies [27,29], which progressively increased the treadmill incline over the sessions.

Five studies [22,28,29,32,33] utilized a comfortable or self-selected gait speed as the basis for determining the training intensity, which was either maintained or increased within or between sessions. In contrast, three studies [31,35,36] adjusted the speed according to the individual’s level of tolerance. Carvalho et al. [25] implemented TT at a speed equivalent to 60% of the estimated maximal oxygen consumption (VO_2_ max), which was calculated using a formula based on the workload during TT or alternatively at 70% of the predicted maximal heart rate based on the participant’s age. This speed was maintained consistently throughout the 30 min session. In contrast, Shulman et al. [27] investigated a high-intensity TT program, commencing at 40–50% of the maximal heart rate reserve and progressing to 70–80%. Conversely, Carda et al. [34] initiated the training program at 80% of the mean speed recorded during the 6 MWT, gradually increasing it to 100%. Picelli et al. [30] maintained a constant speed during the training intervals.

### 4.1. Control Groups

As described in the Methods section, the control interventions were categorized into two types of programs: conventional and robot-assisted.

Conventional programs included strength exercises [25,27,28], overground gait training [24,25,36], walking with RAS [26,33], calisthenics [5,36], stretching and flexibility exercises [27,32], daily activity training [36], proprioceptive neuromuscular facilitation exercises [30], dance [32], and low-intensity exercise combinations [29].

Robot-assisted gait training was conducted using four different robotic systems [30,31,34,35].

Since there were few studies included in the meta-analysis, a separate analysis of the outcomes for the two categories was not performed.

### 4.2. Outcomes

A meta-analysis was conducted on three outcomes: motor symptoms of PD, walking capacity, and functional mobility. Due to the type of intervention and the outcome in the articles, we only performed the statistical analysis on a subset of the included trials.

#### 4.2.1. Motor Symptoms of Parkinson’s Disease

We were interested in studying the impact of TT on quantitative assessments of motor symptoms in PD patients. For this reason, we conducted a meta-analysis on the UPDRS-III scores.

**Figure 2:**
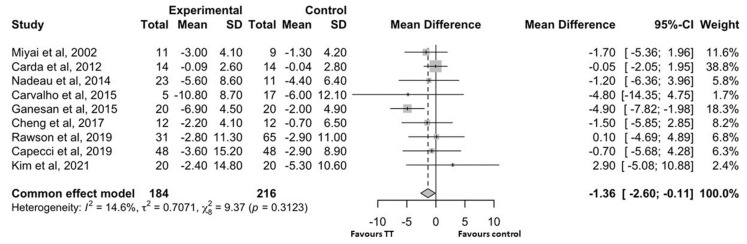
Forest plot for the studies that evaluated UPDRS-III scores. SD: standard deviation; CI: confidence interval [24,25,28,29,31,32,34,35,36].

Figure 2 shows the forest plot for the nine studies [24,25,28,29,31,32,34,35,36] (400 patients: 184 in treatment group and 216 controls) that evaluated motor control using the UPDRS-III. Due to low heterogeneity (I2 = 14.6, *p* = 0.312), we performed a fixed-effect model analysis.

The intervention group showed a slight but statistically significant improvement in motor control (UPDRS-III score) compared to the control group [MD: −1.36 (95% CI: −2.60 to−0.11)]. In 67% of the studies with TT UPDRS-III scores and 33% with control group scores, the mean change in UPDRS-III score was ≥−3.25 points (MCID).

#### 4.2.2. Walking Capacity

**Figure 3:**
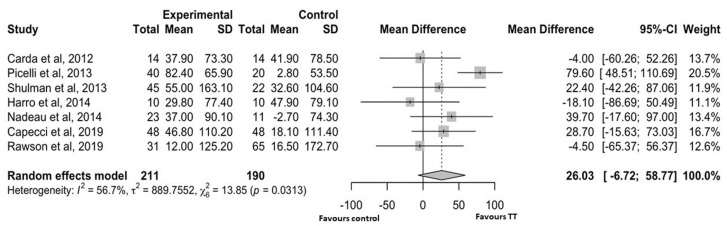
Forest plot for the studies that evaluated the 6 MWT results. SD: standard deviation; CI: confidence interval [26,27,29,30,31,32,34].

To investigate the improvement in walking capacity, we conducted a meta-analysis on the 6 MWT results. Figure 3 shows the forest plot for the seven studies [26,27,29,30,31,32,34] (401 patients: 211 in treatment group and 190 controls) that assessed walking ability using the 6 MWT. Owing to high heterogeneity in the fixed-effect analysis (I2 = 56.7%, *p* = 0.031), a random-effect analysis was performed.

There was no significant difference in the improvement of walking ability between the intervention and control groups [MD: 26.03 (95% CI: −6.72 to 58.77)] despite a tendency toward a stronger effect in the intervention group compared to the control group. In 71% of the studies with TT 6 MWT results and 43% with control group data, the mean change was ≥30.5 m (MCID).

#### 4.2.3. Functional Mobility

To measure the effects of TT on functional mobility, we analyzed the TUG assessment results.

**Figure 4:**
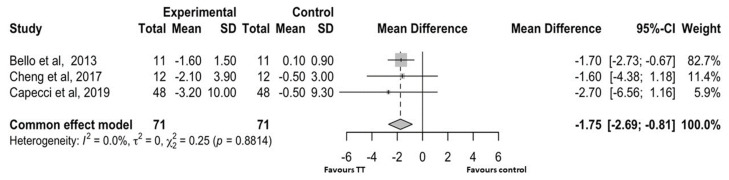
Forest plot for the studies that evaluated TUG test performance. SD: standard deviation; CI: confidence interval [28,31,33].

Figure 4 shows the forest plot for the three studies [28,31,33] (142 patients: 71 in treatment group and 71 controls) that reported variations in the TUG test results. As a non-significant heterogeneity was found (I2 = 0.0, *p* = 0.881), we performed fixed-effect model analysis.

The intervention group showed a significantly higher improvement in TUG test performance compared to the control group [MD: −1.75 (95% CI: −2.69 to −0.81)]. In all studies of TT, TUG test performance improved, and in one study [32], the improvement was close to −3.5 s (MDC). In contrast, in 100% of the controls, TUG performance was unchanged or only slightly changed.

#### 4.2.4. Risk of Bias Assessment

**Figure 5:**
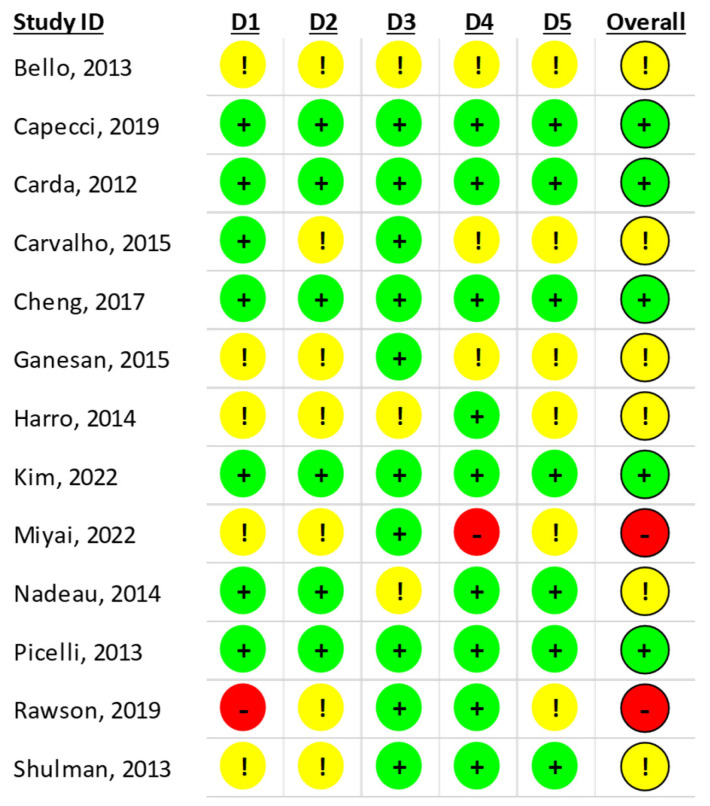
Risk of bias according to the RoB 2 tool [16]. +: low risk; !: some concerns; −: high risk; D1: risk of bias arising from randomization process; D2: risk of bias due to deviations from intended intervention (effect of assignment intervention); D3: risk of bias due to deviations from the intended intervention (effect of adhering to intervention); D4: risk of bias due to missing outcome data; D5: risk of bias in measurement of the outcome [24,25,26,27,28,29,30,31,32,33,34,35,36].

The risk of bias assessment showed an overall moderate quality for the design of the included studies. Figure 5 shows the results of the risk of bias assessment using the RoB 2 tool [16].

Several studies lacked sufficient details about the randomization process. In addition, a significant number of trials reported a high dropout rate, which was not taken into account in the results. Moreover, sample size analysis was not consistently applied, and the assessors were not always blinded, while blinding of the participants and trainers was considered not feasible in all the studies.

## 5. Discussion

Our study suggests that treadmill training is still a cornerstone of gait rehabilitation in Parkinson’s disease (PD). TT appears to be effective in improving the motor symptoms and functional mobility, with significant improvements observed in both Unified Parkinson’s Disease Rating Scale (UPDRS-III) scores and Timed Up and Go (TUG) test performance compared with other types of motor rehabilitation treatment. No superiority was found for walking capacity, as assessed by the 6 MWT.

Gait disturbance induced by Parkinson’s disease is one of the most common gait disorders encountered in clinical practice [37] and is an important contributor to disability and quality of life in mild to moderate PD [38]. Standardization of rehabilitation gait protocols and the search for new strategies, such as robotic and virtual reality gait training, are ongoing [39]. Treadmill training has the advantage that some treadmill programs only require the supervision of a physiotherapist compared with other types of interventions that require the physiotherapist to work directly on the patient.

Our meta-analysis included seven studies published after the 2015 Cochrane review [24,25,26,28,31,32,35] that compared treadmill training with several rehabilitation strategies, with a further 134 patients, including two studies that compared TT with RAGT [31,34], one study that compared TT with Argentine tango with a caregiver [32], and one study that compared TT with calisthenics [25]. This study found that TT seems to produce better effects on functional mobility compared to other forms of rehabilitation treatment. A previous Cochrane review [9] indicated positive effects of TT on walking speed and stride length compared to non-TT interventions, which included undefined or no interventions. This study only selected RCTs with a control group that received an active rehabilitation intervention but still found a statistically significant greater improvement in measures of functional mobility (TUG test).

Regarding the motor aspects of the disease, a previous meta-analysis [7] conducted on a small number of patients (*n* = 16) did not find significant variations in the UPDRS-III score after treadmill training. However, in comparison, the current study used a much larger number of patients (*n* = 184) and demonstrated that patients treated with TT showed a small but significant improvement in motor control (higher UPDRS-III scores).

Studies that considered different rehabilitation techniques have shown mixed results regarding motor control in PD, measured using the UPDRS-III. Carda et al. [34] found that robotic gait training with the Lokomat ^®^ (Hocoma AG, Volketswil [ZH], Switzerland) did not produce significant improvements in UPDRS-III scores. Capecci et al. [31] reported that RAGT resulted in a significant improvement in total UPDRS scores, mainly due to improvements in UPDRS activities of daily living (ADL). Although there was a beneficial effect on gait speed and stride length compared to conventional physiotherapy, Miyai et al. [36] found that there were no significant differences in UPDRS scores and subscores between the intervention and control groups after adjusting for multiple comparisons, while Ganesan et al. [24] showed that the Partial Weighted Supported Treadmill Gait Training (PWSTT) group had a significantly better UPDRS motor score.

The mean improvement in UPDRS-III score in the treadmill group was higher than the MCID in four of the nine considered studies [24,29,31,34]. However, although statistically significant, the average improvement in UPDRS-III score was modest from a clinical perspective as it did not reach the MCID. This may be because the motor subscale of the UPDRS considers several factors, such as facial expression, tremors, and upper limb mobility, which are less affected by TT rehabilitation treatment.

Our results are consistent with previously reported findings [7,9] as we confirmed a significantly greater improvement in TUG performance (a measure of functional mobility) compared to the control group. Despite a significantly greater improvement in patients treated with TT compared to controls in the trials examined [28,31,33], only one study showed an improvement in TUG test performance that was similar to the MDC in patients treated with TT [31]. Since the TUG test requires the patient to stand up, start walking, and change direction, its results are more descriptive and relevant to the daily reality of patients with Parkinson’s disease compared to the simple measurement of walking speed.

One of the main determinants of the longer test execution time in PD patients is the presence of Freezing of Gait (FOG). To date, the effects of rehabilitation on FOG have shown mixed results: a systematic review of rehabilitation interventions showed that FOG leads to impaired mobility and falls, but the effect of rehabilitation interventions on FOG (including physiotherapy and exercise) was uncertain [40]. Other studies have shown that TT is an effective intervention for improving scores on the Freezing of Gait Questionnaire (FOG-Q) [41]. In addition, TUG time has been significantly and independently associated with future falls [42]. For these reasons, even a minimal improvement in TUG test performance with non-pharmacological and cost-effective treatments, such as TT, can reasonably be considered clinically relevant in the context of a progressive neurodegenerative disease. Further studies are needed to determine whether TT is directly associated with a reduction in fall risk. Furthermore, it remains to be determined whether repeated or prolonged treatments over time can produce additional benefits.

Although our meta-analysis failed to highlight a significant difference in the improvement in walking capacity (6 MWT score) between the intervention and control groups, there was a trend towards a greater effect in the intervention group compared with the control group. Indeed, five TT studies out of seven [27,29,30,31,34] showed an improvement in 6 MWT score greater than the MCID. As previously demonstrated [43,44], patients with Parkinson’s disease often report reduced exercise tolerance and severe respiratory muscle impairment. Therefore, a lack of close control over the intensity and volume of exercise and progression (typical, for example, of cardiorespiratory rehabilitation settings) could reduce the effectiveness of TT in tests that include exercise tolerance, such as the 6 MWT.

Furthermore, although not statistically significant, the improvement in 6 MWT scores induced by TT was close to the MCID. It should be highlighted that many of the control interventions (such as Argentine tango and balance and strength exercises) may also increase walking endurance and, therefore, this may narrow the difference in 6 MWT scores when compared to TT. Further studies should consider combining TT with such control interventions in order to enhance walking ability outcomes.

The effectiveness of treadmill training compared to other rehabilitation procedures may have neurobiological bases. In fact, it has been highlighted that in PD animal models, treadmill exercise induces modulation in different brain areas, including the caudate–putamen and the substantia nigra pars compacta, leading to recovery of dopaminergic system function and improvement of motor symptoms [45]. Additionally, a number of beneficial effects of treadmill exercise have been described in animal models: it decreased cell death pathway activity, enhanced neurotrophic factor and mitochondrial functions, increased neurogenesis, and improved synaptic plasticity [45].

A possible explanation for the discrepancy between the different studies regarding the effect of TT on motor aspects of the pathology may be partly due to the fact that the studies enrolled patients at different stages of the disease. In addition, the protocols used for treadmill training (duration, frequency, intensity, and speed) varied between trials.

For this reason, further studies should evaluate the standardization of treadmill rehabilitation programs. In light of the respiratory issues encountered by patients with Parkinson’s disease, it could be interesting to consider integrating respiratory physiotherapy and 6 MWTs into treadmill rehabilitation programs.

### Limitations

Although the methodological quality of the included RCTs was generally moderate, some potential limitations of trials investigating TT need to be acknowledged, such as the inability to blind the participants and the physiotherapists who delivered the training.

It should also be emphasized that the included RCTs did not enroll patients with advanced Parkinson’s disease (Hoehn and Yahr stage 4); therefore, the results of this study can only be generalized to patients in mild to moderate stages of the disease (Hoehn and Yahr stages 1–3). The authors also acknowledge the possibility of publication bias, as they did not extensively search the gray literature.

## 6. Conclusions

In conclusion, our meta-analysis confirmed that treadmill training is an effective rehabilitation strategy for improving gait and mobility in PD patients and highlights the potential of including it in all PD rehabilitation programs. Furthermore, we showed that TT can provide benefits over other active treatments, including robotic gait training and unconventional approaches, such as dance (e.g., tango) or calisthenics.

## Figures and Tables

**Table 1:** Baseline characteristics of participants in the included studies.

Characteristic	No. of Studies (No. of Participants)	Pre-Intervention Mean
Age, years	13 (565)	65.41 ± 3.62
Male, *n* (%)	12 (531)	315 (59.3%)
Height, cm	7 (274)	165.43 ± 4.65
Weight, kg	6 (252)	68.11 ± 8.9
Duration of the disease, years	12 (531)	5.96 ± 1.72
Mini Mental State Examination score	8 (389)	27.32 ± 1.22
Hoehn and Yahr scale score	13 (565)	2.35 ± 0.42
UPDRS-III score	11 (517)	26.88 ± 7.65

UPDRS: Unified Parkinson’s Disease Rating Scale. Data are expressed as mean ± SD, unless stated otherwise.

**Table 2:** Details of the studies included.

Ref.	H&Y Inclusion	H&Y Score, Mean ± SD	UPDRS- III Score	Disease Duration, Years	Program Duration, Weeks	Dropouts, *n*	Participants, *n*	Intervention and Control	Intensity of TT	Frequency of TT	Duration of TT	Setting
Ganesan 2015 [24]	Stage ≤ 3	2.08 ± 0.18	30.93 ± 1.48	5.4 ± 1.56	4	0	20 20	**Intervention:** Partial weight-supported treadmill with unweighting support system (±20%) and visual monitoring of step length **Control:** Conventional gait training with verbal cues and swinging strategies + non-intervention group ± excluded	3 sets of 10 min training interspersed with 2 min of rest, at comfortable speed initially, with progressive increase to fast comfortable speed	4 times/week	5 min warm up + 30 min training + 5 min cool down	Outpatient
Carvalho 2015 [25]	Stages 1–3	2.3 ± 0.35	36.01 ± 6.32	5.63 ± 1.49	12	0	5 8 9	**Intervention:** Treadmill training **Control:** (A) Strength exercises for large muscle groups (B) Calisthenics exercises and gait training	60% of estimated VO_2_ max or 70% of predicted HR max	2 times/week	5 min warm up + 30 min training + 5 min cool down	Outpatient
Harro 2014 [26]	Stages 1–3	1.93 ± 0.57		4.12 ± 2.26	6	1 1	10 10	**Intervention:** Speed-dependent treadmill training **Control:** RAS during ground walking using headphones with a personalized music playlist at bpm based on comfortable gait speed, which was progressively increased	Three 5 min intervals: 1st and 2nd at maximal walking speed, 3rd at 5% more than maximal walking speed, interspersed with 2.5 min at comfortable gait speed	3 times/week	5 min warm up + 20 min training + 5 min cool down	Unknown
Shulman 2013 [27]	Stages 1–3	2.18 ± 0.36	32.1 ± 9.9	6.2 ± 3.8	12	3 4 5	23 22 22	**Intervention:** (A) High-intensity treadmill (B) Low-intensity treadmill **Control:** Stretching and lower limbs resistance training	Initially 15 min at 40–50% of the maximal HRR, increased by 0.2 km/h every 5 min (increased by 1% every 2 weeks) until 30 min (70–80% of HRR). Initially 15 min at 0% incline and self-selected pace, increased by 5 min to reach 50 min	3 times/week	30 min high-intensity training, 50 min low-intensity training	Community-based?
Cheng 2017 [28]	Stages 1–3	1.9 ± 0.47	19.6 ± 2.19	7.1 ± 1.78	4–6	0	12 12	**Intervention:** Turning-based curved treadmill **Control:** Trunk and upper limb exercises + both groups also performed 10 min of overground gait training	15 min clockwise and 15 min counterclockwise at 80% of comfortable walking speed initially, progressively increased by 0.05 m/s every 5 min as tolerated	2 or 3 times/week	30 min	Unknown
Nadeau 2014 [29]	Stage ≤ 2	1.91 ± 0.25	23.15 ± 4.28		24	17 18 23	12 11 11	**Intervention:** (A) Speed treadmill (B) Mixed treadmill **Control:** Low-intensity exercise routine ± tai chi, dance, coordination, and resistance band exercises	80% of preferential walking speed, progressing to 90–100% with speed increased by 0.2 km/h each session 80% of preferential walking speed, progressing to 90–100% with alternating increase in speed of 0.2 km/h or in incline of 1% each session	3 times/week	5 min warm up + 45 min training + 5 min cool down	Outpatient
Picelli 2013 [30]	3	3	18.02 ± 1.53	6.77 ± 5.8	4	0	20 20 20	**Intervention:** Treadmill **Control:** (A) RAGT with GT1 and partial body weight support (B) Conventional gait training with proprioceptive neuromuscular facilitation	3 sets of 10 min at speeds of 1, 1.5, and 2 km/h, respectively, interspersed with 5 min of rest	3 times/week	45 min	Outpatient
Capecci 2019 [31]	Stage ≥ 2	3 ± 0.74	23.65 ± 2.77	8.9 ± 4.8	4	2 12	48 48	**Intervention:** Treadmill **Control:** RAGT with G-EO and partial body weight support at increasing speed	Initial speed of 0.8–1 km/h, gradually increasing to 2.0 km/h or higher depending on tolerance	5 times/week	45 min	Outpatient
Rawson 2019 [32]	Stages 1–4	2.18 ± 0.52	36.69 ± 11.19	5.47 ± 4.59	12	12 7 12	29 36 23	**Intervention:** Treadmill **Control:** (A) Argentine tango with a caregiver (B) Stretching and flexibility whole-body exercises	Preferred walking speed	2 times/week	60 min with minimal warm up and cool down	Community-based
Bello 2013 [33]	Stages 1–4	2.16 ± 0.20	20.36 ± 3.73	4.89 ± 1.26	5	Unknown	11 11	**Intervention:** Treadmill **Control:** Overground training with RAS	4 bouts of 4 min of walking at preferred speed, interspersed with 3 min of rest, increasing by 4 min every week	3 times/week	25 min initially, increasing to 40 min	Unknown
Carda 2012 [34]	Stage < 3	2.2 ± 0.5	10.53 ± 4.04	3.73 ± 0.25	4	1 1	14 14	**Intervention:** Treadmill **Control:** RAGT with Lokomat and partial body weight support	Initial speed at 80% of the mean speed of the 6 MWT, progressively increasing to 100% in the last two weeks of training	3 times/week	30 min	Outpatient
Kim 2022 [35]	Stages 2.5–3	2.8 ± 0.24	36.6 ± 3.58	9 ± 1.56	4	2 2	20 20	**Intervention:** Treadmill **Control:** RAGT with Walkbot-S and visual feedback and auditory cues at the toe-off phase to improve the rhythm of the gait + contemporary rehabilitation treatments allowed in both groups	Initial speed based on subjects’ height and gradually increasing throughout the sessions to the maximal velocity, depending on tolerance	3 times/week	15 min warm up and cool down + 30 min training	

H&Y: Hoehn and Yahr scale; VO_2_ max: maximum rate of oxygen consumption; HR: heart rate; HRR: heart rate reserve; RAGT: robot-assisted gait training; GT1: Gait Trainer GT1 (Reha-Stim, Berlin, Germany); 6 MWT: 6 min walking test; G-EO: G-EO system (Reha Technology AG, Olten, Switzerland); RAS: rhythmic auditory stimulation. The control and intervention groups are shown in bold.

## Data Availability

Data are available in the paper and Supplementary Materials or from the corresponding author.

## Supplementary Materials

The following supporting information can be downloaded at https://www.mdpi.com/article/10.3390/brainsci15080788/s1, Table S1: Supplemental Material_ROB2_EB; Table S2: Supplemental material ROB2_CS.
