# Supplementary material for: Treadmill Training in Patients with Parkinson’s Disease: A Systematic Review and Meta-Analysis on Rehabilitation Outcomes
Source: Brain Sci. 2025 Jul 24;15(8):788. doi: 10.3390/brainsci15080788 (PMC12384747; doi:10.3390/brainsci15080788)
Supplement: Supplementary file 1 [file brainsci-15-00788-s001.zip › brainsci-3732878 -Supplementary Materials.pdf]

Systematic Review and Meta-analysis

TREADMILL TRAINING IN PATIENTS WITH PARKINSON'S DISEASE: A SYSTEMATIC REVIEW AND META-ANALYSIS ON REHABILITATION OUTCOMES

Supplementary Materials

Table S1: Supplemental material ROB2_EB.

Table S2: Supplemental material ROB2_CS.
